# Cremation

**Published:** 1885-06

**Authors:** J. G. Davey


### CREMATION.

BY

J. G. Davey, M.D., Clifton.

*(Read at meeting of Bristol Medico-Chirurgical Society.)*

---

If, as has been said, "*Progress is the law of Life,*" and if this same "progress" is seriously obstructed by the ignorance of the public at large—its want of information in so far as the laws of health and the sources of disease are concerned—and if, moreover, we, as members of the medical profession, are cognisant of these same laws, and can and do appreciate the many and ever-active sources of suffering and disease among our kind, does it not follow that we are under a deep obligation to teach the public (as best we can) the many means whereby the laws alluded to above may be duly obeyed, and the health preserved; or, what is the same thing, much suffering and disease averted, or, to say the least of it, very materially lessened? I cannot doubt that our duty goes far, very far, in the direction here indicated.

To this present time we have worked fairly well, as teachers of hygiene; but now it must be admitted that the world is more than ever astir. It has, it would appear, crept well out of its shell, and far into the sunshine of a higher life; but we must still keep the lead. Our advantages, educational and practical, over the masses of mankind are so certain and demonstrable that—to use the significant and telling phrase of a late President of the American States, the famous Lincoln—we are bound to "KEEP PEGGING AWAY."

Now, there is no single question agitating the public mind more than that of the disposal of our dead. To within the last year or two, cremation hardly stirred the feelings of the most sensitive. It is to this very important subject that I desire to call the attention of those present this evening. I hope to convince you that, regarded from a sanitary point of view, the burning of the dead is most desirable, and is, in all respects, to be commended. In view to the entirety of the subject—its rise and progress to this present time—it may be well, perhaps, to refer to the labours, commenced now forty years since by a London surgeon—the late G. A. Walker. Though a hard-worked practitioner, he failed not to make his profession and the public his debtors through time for his very clever and thoroughly practical book, entitled, *Gatherings from the Graveyards of London.*

The prevention of disease was, indeed, Walker's speciality: to this great end he devoted the best years of a well-spent life. With the conviction that "burial-places in the neighbourhood of the living are a national evil"—"the harbingers, if not the originators of pestilence"—"the cause, direct or indirect, of inhumanity, immorality, and irreligion;" "that some of the most afflictive visitations of Providence have originated in the contaminations of the atmosphere from putrefying animal substances;" and that "to the neighbourhood of the graveyard may be attributed the violence, if not the origin, of some of the most destructive diseases which have depopulated the human race"—he (Walker) then offers a meed of praise to America for its efforts towards the substitution of the "*cemetery*" for the aforesaid "*graveyard*," and adds these words—words pregnant then, as now, with warning to the party who would "*rest and be thankful*"—viz., "But England looks on, a silent and unmoved spectatress of some of the most offensive and dangerous encroachments upon the security and sanctity of the resting-places of her dead."

Walker's book here quoted is thoroughly practical—the outcome of investigations carried on during a series of years, and embracing a close examination of some three dozen of the then "Metropolitan burying-places."

The Secretary of State of the time alluded to was unmoved in the matter, and to Walker's "copious general statements no kind of acknowledgment was made." But the Press, we are told, "was at length attracted to the very flagrant abuses of intramural interment, and hence, and after long time, the erection of cemeteries—such as now exist."

Time, however, moves on, and with it circumstances change; new wants arise. With the progress of medical science, the comparatively rough-and-ready means to hand, of a past time, are found insufficient and defective. Furthermore, the enormously increased and increasing population here and everywhere makes its demands on the attention of the thinking members of society. Such demands must be responded to. In the days of Walker it was held sufficient to recognise the fact that "those who dig graves do not live long;" that "the vapours which they respire soon destroy them;" that "animal exhalations, and especially those which arise from a dead body in the process of putrefaction, are very afflicting and very dangerous;" that "the peculiar character and virulence of disease in the proximity of graveyards" was a matter of demonstration; and that the "miasmata from animal putrescency may and do occasion not only the instantaneous loss of human existence, but increase the intensity of pestilential diseases." But such hard facts, though of deep and serious import, cannot be said to tell all the truth, in this year of grace 1884! As our late President (Dr. Fyffe) told us in his Inaugural Address of last year: "We live now in days when busy brains are searching with all their powers for the ultimate causes of specific diseases; when the investigation for living organisms in the blood and animal tissues has become the absorbing object of modern pathological inquiry." As you know well, Tyndall confirms the above position. In words ever to be remembered he has declared that "the physician and the sanitarian have no longer to fight against phantoms—their enemy is revealed." It is not at this time as it was when Walker wrote: the contention now is something more than the mere noxious gases, but "*organised germs.*" Contagia are living things. Men and women have died by thousands, and are now dying by thousands, that bacteria and bacilli might live. These—the outcome of modern or recent investigation, and the sources at once of disease and death—demand something more than Walker advocated, *i.e.*, a remedy more potent than any he conceived. Our only recourse is *cremation*. The burial-places of this day—the cemeteries—have become, what Walker described the old graveyards; the first-named, or many of them, are no longer suburban—really suburban—but enclosed by the habitations of the living. The increased and increasing population everywhere must add to the value of land. Our present dependence on America, and on the Continent and elsewhere, for food must tend to increase this value. The land must become really utilised; must be devoted to its legitimate purposes—the growth of food—and not to the burial of the dead.

In an able and instructive tale from the pen of the Rev. H. R. Haweis, entitled, *Ashes to Ashes: a Cremation*

*Prelude*, we read thus: "Go through London, and many another large town, once a rural hamlet, now a *crowded city, with a pest-house in its centre.* That is the history of your burial-grounds; that in time must be the history of all burial-grounds," by whatever names recognised. "Look," it is added, "at your Brompton, your Kensal Green, your Norwood cemeteries and others. But yesterday in open fields, now the centres of a growing population; now fast filling—nay, full to repletion." The cemetery of to-day *is* fast becoming—as a consequence of the overcrowding just alluded to—a real centre for much the same kind of "*management*" as did the old grave-yard. Mr. Haweis writes: "If grave-diggers have ever broken off the plates from coffins, if they have broken up coffins before they were decayed, if they have removed bodies to the bonehouse before they have been sufficiently decayed to make the removal decent—what surety have we that they do not, nor will not, repeat the abominations? None at all. But grave-diggers are not, it appears, alone to blame. The objections so manifold in the present mode of disposal of our dead have other sources. In spite of our Government Inspectors and our Boards of Health, the overcrowding of the churchyard and the cemetery is of no infrequent occurrence. The mould in many a burial-place, it is well-known, is literally saturated with the dead; and in spite of such a state of things, the hearses stream in to deposit their ghastly freights of human merchandise. The burial fees are not without their attractions, notwithstanding the pressure in regard to space and overcrowding." It may be said that such a mode of business or "*management*" as is here referred to belongs to the past—has ceased to be; that the grave-digger, and not less the capitalist, are now under the influence of better and loftier principles of conduct; but the annexed paragraph, which I have taken from the *Scotsman* newspaper but a comparatively short time since, will show that such "*management*" has not yet ceased to be, and that the same filthy love of gain or lucre still holds the higher, the nobler, faculties of man in abeyance.

In the *Scotsman*, then, are seen these words; viz., "The burial-ground of St. Cuthbert is situate in the centre of Edinburgh; it is one of the oldest. In the last fifteen years 10,800 bodies have been placed there. Graves were reopened up to 1874; some every seven, some every three years. Coffins with no plates were usually broken open; the remains were often not 'ripe'—that is, undecayed—the coffin-wood was burned, the remains heaped pell-mell upon others, to make room for more. At last, but one or two inches of earth could be placed between the coffins. When all this came out in court, the sheriff observed that, although such revolting practices were freely carried on in other churchyards, there was no reason why they should continue in the heart of Edinburgh." Yes, the remark of the sheriff was, and is now, true to a large extent. "Revolting practices," of a kind, are rife now at the cemeteries at Highgate, Kensal Green, and Brompton, among others. These burial-places, it should be known, were condemned, in 1850, by the Board of Health, and ordered to be closed, in the interests of the public health; but to this day they remain *unclosed*. The last-named cemetery was bought by Parliament, yet the Government of the country is now using it, "at high pressure and remunerative rates," though "condemned" by its own Board a generation since!

Under the circumstances narrated, how little are the relics of the dead respected in this year of grace, 1884! How little indeed, when we learn from the *Quarterly Review* (No. 42, p. 380) that "many tons of human bones every year are sent from London to the North, where they are crushed in mills constructed for the purpose, and used as manure!" Well may the Rev. H. R. Haweis exclaim, or rather write: "Truly the unclean monster of burial has been too long on trial. He has been tried and found guilty of murder, robbery, and every kind of corruption. *The pure fire-angel of Cremation stands now at the door.*" I will now ask your attention to the various and specific forms of danger and disease of which the disposal of the dead by fire would relieve the living, and to other advantages to be gained by cremation.

To the plea for cremation from the facile pen of the Rev. John Constable, rector of Marston-Biggott, near Frome, I would invite your attention. The clergyman named officiated at the cremation of the body of the late Captain Hanham, to the memory of whom, as well as to that of the late Lady Dilke, be all honour. They offered a bright example to the living, one which WE, Mr. President, will do well to follow. Mr. Constable writes thus; viz.: "The account of the cremation of the body of the late Captain Hanham has greatly revived the public interest in that method of disposing of our dead. I venture, therefore, to hope that you will insert in your journal" (the editor of a local daily paper is being addressed) "an important extract, which bears directly on the question. In the reports from Her Majesty's Diplomatic and Consular Offices abroad on subjects of general interest, presented to both Houses of Parliament this year, there is one from Mr. Corbett, of Rio de Janeiro, embodying the investigations of Dr. Freire on the subject of yellow fever. Dr. Freire writes: 'I think it a duty to divulge as soon as possible a circumstance of much importance to the public health. Having gone to visit the Turnjuba cemetery, where those dying in the Maritime Hospital of Santa Isabel are interred, I gathered from a foot below the surface some of the earth from the grave of a person who died about a year ago of yellow fever. On examining a small quantity with the microscope, I found myriads of microbii exactly identical with those found in the excreta of persons sick with yellow fever. These observations, which were verified in all their details by my auxiliaries, show that the germs of yellow fever perpetuate themselves in the cemeteries, which are like so many nurseries for the propagation of new generations destined to devastate our city. A guinea-pig, whose blood, examination showed, was in a pure state, was shut up in a confined space, in which was placed the earth taken from that grave. In five days the animal was dead, and its blood proved to be literally crammed with disease germs in various stages of evolution.' "

Side by side with the experiences of Dr. Freire, of Santa Isabel, we have those of the famous Pasteur, and from which it appears that to the common earthworm must be referred the occurrence of anthrax or splenic fever in cattle and sheep.

It appears, then, that in CREMATION alone can be found the necessary protection from "disease germs" of whatever kind, or by whatever names recognised—"Bacteria" or "Bacilli," and so on. It must be borne well in mind that both the air we respire and the water we drink are utilised by such germs—that is to say, are made subservient to them—and hence, in great part, the fearful diffusion of disease and death.

The late Dr. Snow proved, as you know, that an outbreak of cholera, in 1854, was due to the use of the water of a particular pump in Soho. This water was said to be contaminated; but with what, it was unknown. Others—among them, Dr. Carpenter—inclined to the opinion that the epidemic was due to "*defiled air*." But, judging from the recent experiments of Dr. Koch at Calcutta, cholera, like yellow fever and splenic fever, is due to "bacilli" of a peculiar form. We may well exclaim, What a revolution is here! What a change in practical medicine has this microscope created! It may well be asked, What may not be its future discoveries in the cause of science, in the interests of mankind?

However, so far as cholera is concerned, we seem bound now to prefer the evidence of Koch to the suggestion of Dr. Carpenter, made in 1854, as to the presence of "defiled air" as the first cause of cholera—let it occur either "in Soho" or within the precincts of a populous Eastern city like Calcutta.

Now, these "*bacilli of a peculiar form*," it is well to remember, are said to be found "in cholera patients only; always in them, and never in other patients." Such was the conclusion of Pasteur to within the last four months, and this was then declared to rest on the most satisfactory evidence. The following extract is from a paper, *On Cholera*, published on the authority, as it would seem, of Pasteur, in the spring of this year, 1884: "The final proof, however, in the actual production of the disease by the administration of the bacillus was wanting. It was given to mice, cats, and dogs, but did them no harm. Dr. Koch, however, made another step. An outbreak of cholera in Calcutta led to the examination of the reservoir or pond from which the people of the stricken district drank, and the cholera bacillus was found in it in great numbers. It was noted, moreover, that as the epidemic died away, there was a clearing of the water from the parasite. This final proof of the discovery is due to Dr. Vincent Richards, an English surgeon in India, who gave some of the bacilli to a pig, which was seized with violent cholera, and died in three hours. This experiment seems to be conclusive, and the discovery may now be regarded as complete." Thus far it would appear that man is very clearly at the mercy of the many kinds of germs revealed to him by scientific research. To this time, he has put his faith, but too exclusively, in antiseptics. With these he has fought the germs so devoted to his destruction and death. Can it be doubted that the most potent means of contention have been to this time unemployed? I allude to CREMATION. To this we must come; it is a real necessity—the one resource altogether inevitable, if we would, as above remarked, "relieve the living of much suffering and disease, and so add to the advantages and blessings of this life."

Now, we have all of us heard much of the extinction of infectious diseases, on the ground that such diseases never arise spontaneously. The assumption is, that small-pox, measles, scarlet fever, whooping-cough, typhus and typhoid fever, do invariably "*breed true;*" or, what is the same thing, neither one produces another of them. For example sake, the virus of small-pox never causes typhus, and so on to the end. If, then, the diseases named are simply contagious, we may hope that in the long run they may be prevented or exterminated. But the mode of their extinction—if there be such a mode—must be such as will take in the dead as well as the living. The per-centage of deaths from the diseases just named, as well as from diphtheria, glanders, malignant pustule, &c., to say nothing of the principal contagious disorders of the domestic animals, cannot be said to be else than high. If, then, as it is believed, each one of the human family, and each and every animal, whose death is caused by either one of the infectious diseases named above, furnishes a centre—the abode of bacteria and such like, seeds whose presence and germination are ever on the alert to infest the healthy and living—what else than the burning of the dead man and animal can be resorted to, or can be relied on to insure their extinction? If such centres—as I have called the dead bodies of those, the prey of the several diseases named—are buried after the old fashion, each one of such can but add to the probabilities of poisoning the spring or watercourse, which may find its exit in some locality fated to suffer therefrom, or, in the due course of time, to add to the heavy cloud of miasma which ever and anon presses on all places of interment of the dead, whether the dead be human or animal. Furthermore, it must be borne in mind that the clothing—the shroud, or what not, about the dead bodies of those dying from fever, diphtheria, cholera, &c., &c., do long retain their virulent properties—a cloak, for instance, having been known to give scarlet fever after having been laid by for eighteen months; and the poison of anthrax or splenic fever of cattle having been found active after keeping for four years; so that if we would realise or bring about the extinction of infectious diseases, those dying from them must be the subjects of the "*pure fire-angel of cremation*," so called by the Rev. H. R. Haweis.

Cremation has many side or indirect relations to medical science; one such is seen in that peculiar condition of the nervous system recognised by the term TRANCE. Now, the occurrence of trance is not so infrequent as some may suppose. In my own practice—covering now a period of more than fifty years—I have seen five such cases. I think it not unlikely that the patient in a trance-fit may be looked on as in a state analogous to that of certain of the lower animals—as the dormouse and bat, &c.—seen in the condition of *hybernation*, when the cerebro-spinal system is at rest, or, for the time being, suspended. In the ordinary and complete trance, as it occurs to man or mankind, the disuse of food (fasting) is commonly present. Trance, it may be added, occurs for the most part spontaneously—is subjective; but I have known it to exist as an objective condition, and to have been induced both by the inhalation of chloroform and by mesmeric passes. Now, the occurrence of trance suggests the idea—and a most painful idea it is—that a patient so afflicted may be either buried or cremated before the life is extinct. But the fact tells nothing as against cremation, but the reverse, as I shall hope to prove. However, in order to prepare the way for such proof, let me quote in detail the particulars of certain cases of trance, if it be only to excite in the minds of my hearers the real necessity which exists for patience and caution in matters pertaining thereto.

I have before me the particulars of four cases of trance. The first was so long as 28 weeks "*in a state of profound sleep*," and during the period named "*she never woke once, nor received nourishment of any kind*." She "*quite recovered*."

In the second case "trance" succeeded to an illness. It lasted "*eight months*." "About once in six weeks," she awoke "during a few hours," and partook "of nourishment of the lightest description," being then "in entire possession of all her faculties." In this instance also recovery took place.

The third instance of the kind occurred at Hereford some two or three years ago. It is headed a "*Narrow Escape of being Buried Alive*," and is described thus; viz: "An extraordinary case of trance has just been brought to light at Hereford. A girl named Sarah Ann Dobbing, eleven years of age, who has lived since she was very young with her great-aunt, Mrs. Derry, was for some days past considered to be dying, and on Tuesday night she was laid out for dead. Arrangements were made for the funeral, and shortly before its appointed time a Miss Cooke and a Miss Bethell came to look at the supposed corpse, and to the amazement, and almost horror, of both, the covering of the body was observed to be moving. The child was then found to be alive, and medical assistance was at once procured. About three weeks before her supposed death the girl's condition underwent a marked change, and for some time she had not been able to take anything but a very little water, passed between her teeth with a teaspoon. Dr. Whitfield and Dr. Smith, who were called in after her revival, had a yolk of a newly-laid egg beaten up, and introduced by means of a syphon. This had an immediate effect on the heart and brain, but it was not long before the stomach returned it. On Friday morning, the patient was in an absolutely unconscious state—the mouth wide open, the tongue protruding its full length, the eyes fully open, the eyeballs turned up and rigidly fixed, and the right hand raised, and the whole frame in a state of hysterical agitation. The patient has been sensibly affected by the alternations of day and night, sleep coming to her aid as the days have closed, and only leaving her as morning arrived. An experienced nurse, who has been much with the child during the last month, had the charge of her when she was assumed to have been dead, and arranged the laying out."

But all the subjects of the "TRANCE-STATE" do not regain their normal state of being—do not recover; and for this simple reason—their real condition, their temporary and abnormal existence, is mistaken for death: they are buried alive.

In the London *Daily Times*, dated May, 1874, you may read the following words: "In August, 1873, a young lady died not very long after her marriage. Within a year the husband married again, and the mother of the first bride resolved to remove her daughter's body to her native town. They open the vault, and find the poor creature's body fallen prostrate on the ground, her hair dishevelled, and her shroud torn to pieces. She had risen out of her trance, and had burst her narrow prison" —her coffin—"but to find herself in one but a trifle larger—but far more inexorable. It must not be supposed," it is added, "that this is altogether exceptional, for at Sonnens, Lower Garonne, and at Bergarac, Dordogue, cases of premature interment have also, and within a few years, occurred; the particulars of these are to be found in the *Sunday Times*."

Now, in so far as trance-patients are concerned, such, it may be said, may be by possibility either buried alive or burnt alive; but if a comparison may be made between these two forms or modes of killing—so to put it—then the comparison must be in favour of CREMATION. Sir Henry Thompson has written on this matter in these words—words so pregnant with truth, and so conclusive, that I hesitate not to call your especial attention to them:

"There is a source of very painful dread—little talked of, it is true, but keenly felt by many persons at some time or another—the horror of which to some is inexpressible. It is the dread of a premature burial; the fear lest some *deep trance* should be mistaken for death, and that the awakening should take place too late. Happily such occurrences must be exceedingly rare, especially in this country, where the interval between death and burial is considerable, and the fear is almost a groundless one. Still, the conviction that such a fate is possible, which cannot be altogether denied, will always be a source of severe trial to some. *With cremation no such catastrophe could ever occur; and the completeness of a properly conducted process would render death instantaneous and painless, if by any unhappy chance an individual so circumstanced were submitted to it.* But the guarantee against this danger would be doubled, since inspection of the entire body must of necessity immediately precede the act of cremation, no such inspection being possible under the present system."

But from whatever standpoint we look at cremation, its great advantages over the present means of disposal of our dead must be evident. Now, there are 100,000 deaths yearly in London, and all these bodies may be said to be buried in the surface-soil around it (London); that is to say, during a period of thirty years there are 3,000,000 deaths. According to Sir Spencer Wells, a body becomes clay after twenty years' burial. If this be the case, there are always 2,000,000 bodies under the feet of those resident in the Metropolis and its suburbs, undergoing putrefaction, or, as he calls it, "*harmful decay.*" If, then, as Dr. Lyon Playfair affirms, the earth "above the dead" is not sufficient to absorb the full amount of the putrid gases evolved, it must follow that the air breathed is highly deleterious, and but too likely to add to and intensify the diseases allotted to man. Given the number of deaths at 100,000 yearly, in and near London, it is estimated that the amount of deleterious gases emitted by such a mass of putrefying bodies as that named above, is 5,140,160 cubic feet, the whole of which—bear in mind —beyond what is absorbed by the soil, must pass either into the water below or the atmosphere above.

To sum up, in the words of Sir Henry Thompson: "For the purposes of cremation nothing is required but an apparatus of a suitable kind, the construction of which is well understood and easy to accomplish. With such apparatus the process is rapid and inoffensive, and the result is perfect. The space necessary for the purpose is small, and but little skilled labour is wanted.

"Not only is its employment compatible with religious rites, but it enables them to be conducted with greater ease and with far greater safety to the attendants than at a cemetery. For example, burial takes place in the open air, and necessitates exposure to all weathers; while cremation is necessarily conducted within a building, which may be constructed to meet the requirements of mourners and attendants in relation to comfort and taste.

"Cremation destroys instantly all infectious quality in the body submitted to the process, and effectually prevents the possibility of other injury to the living from the remains at any future time. All care to prevent such evil is obviously unnecessary, and ceases from the moment the process commences."

In a word, the aim of cremation is to prevent the process of putrefaction in the dead, whilst it conserves the health of the living.

